# The *Glycine max* cv. Enrei Genome for Improvement of Japanese Soybean Cultivars

**DOI:** 10.1155/2015/358127

**Published:** 2015-06-23

**Authors:** Michihiko Shimomura, Hiroyuki Kanamori, Setsuko Komatsu, Nobukazu Namiki, Yoshiyuki Mukai, Kanako Kurita, Kaori Kamatsuki, Hiroshi Ikawa, Ryoichi Yano, Masao Ishimoto, Akito Kaga, Yuichi Katayose

**Affiliations:** ^1^Mitsubishi Space Software Co., Ltd., Takezono, Tsukuba, Ibaraki 305-0032, Japan; ^2^National Institute of Agrobiological Sciences, Owashi, Tsukuba, Ibaraki 305-8634, Japan; ^3^NARO Institute of Crop Sciences, Kannondai, Tsukuba, Ibaraki 305-8518, Japan; ^4^University of Tsukuba, Tennodai, Tsukuba, Ibaraki 305-0006, Japan; ^5^National Institute of Agrobiological Sciences, Kannondai, Tsukuba, Ibaraki 305-8602, Japan

## Abstract

We elucidated the genome sequence of *Glycine max* cv. Enrei to provide a reference for characterization of Japanese domestic soybean cultivars. The whole genome sequence obtained using a next-generation sequencer was used for reference mapping into the current genome assembly of *G. max* cv. Williams 82 obtained by the Soybean Genome Sequencing Consortium in the USA. After sequencing and assembling the whole genome shotgun reads, we obtained a data set with about 928 Mbs total bases and 60,838 gene models. Phylogenetic analysis provided glimpses into the ancestral relationships of both cultivars and their divergence from the complex that include the wild relatives of soybean. The gene models were analyzed in relation to traits associated with anthocyanin and flavonoid biosynthesis and an overall profile of the proteome. The sequence data are made available in DAIZUbase in order to provide a comprehensive informatics resource for comparative genomics of a wide range of soybean cultivars in Japan and a reference tool for improvement of soybean cultivars worldwide.

## 1. Introduction

Soybean (*Glycine max*) is one of the world's most important leguminous crops being a major source of edible proteins and vegetable oils. In terms of global production, soybean ranks fourth, following the major cereal crops such as rice, wheat, and corn. It is also a major source of nutritionally and physiologically active substances such as saponins, isoflavones, phytosterols, and tocopherols. Consumption of soybeans as food is largely concentrated in Asia. Soybean has been a part of the Japanese diet and eaten from ancient times as a valuable source of traditional fermented products such as miso, soy sauce, and natto and nonfermented products such as edamame (boiled soybean), kinako (toasted soybean flour), tofu, and soymilk. As in other major crops, the main targets of soybean breeding in Japan are high yield, high quality (absence of seed coat cracking, seed size, hilum color, uniformity of seed size, and food processing adaptability) to compete with imported soybean, and resistance to biotic/abiotic stress for stable production. Additionally, the chemical component of seeds including high protein content, modification of storage proteins, absence of lipoxygenases and saponin, high isoflavone content, and high sucrose have been given much consideration in many soybean breeding programs [1].

The domesticated soybean has its origin from* Glycine soja,* a wild soybean species found mainly in northern China, Japan, Korea, and the eastern part of Russia [2]. Archaeological studies indicate that the word for soybean first appeared in China about 3,700 years ago in bone inscriptions dating back to the Yin and Shang dynasties and carbonized soybean seeds found about 2,600 years ago [2]. Estimation of archaeological records indicates a widespread early association of small seeded soybean to be as old as 9,000–8,600 calibrated years before the present (cal BP) in northern China and 7,000 cal BP in Japan [3]. Direct radiocarbon dates on charred soybean seeds indicate selection resulted in large seed sizes in Japan by 5,000 cal BP (Middle Jomon) and in Korea by 3,000 cal BP (Early Mumun) [3]. Extensive genome analysis also indicates that the* G. soja*/*G. max* complex diverged from the most recent common ancestor at 0.27 Mya [4] or 0.8 Mya [5]. In a more recent study, the genetic variation and population structure among 1,603 soybean accessions indicated a clear genetic differentiation among Japanese soybean landraces, exotic and cultivated soybeans, and wild soybeans [6].

From the genomics point of view, soybean has been used as a model plant for comparative studies of legumes in terms of root nodulation, oilseed production, and secondary metabolism. It is also a valuable material for genome research because of the availability of many genomic and germplasm resources. In 2010 a great deal of effort in the USA culminated with the sequencing of the paleopolyploid soybean genome based on a soybean cultivar Williams 82 [7]. This cultivar was derived from backcrossing a* Phytophthora* root rot resistance locus from the donor parent Kingwa which was selected in 1921 from the cultivar Peking introduced from Beijing, China, in 1906 [8].

In Japan, however, domestic cultivars have been developed to suit a variety of conditions and applications of specific importance to Japanese growers. Although the* G. max* cv. Williams 82 reference soybean genome sequence could be useful in understanding the diversity among many cultivars, it is necessary to have genomic resources that could be directly applied to Japanese soybean cultivation. The Japanese soybean cultivar Enrei was derived from cultivars Norin number 2 and Higashiyama number 6 (also known as cv. Shiromeyutaka) and was developed in 1971 at Kikyogahara Branch of the Nagano Agricultural Experiment Station (presently known as Nagano Vegetable and Ornamental Crops Experiment Station) [9]. In this paper, we described the analysis of the genome sequence of the Japanese soybean cultivar Enrei focusing on the phylogenetic analysis and major traits for soybean breeding including anthocyanin and flavonoid biosynthesis and proteome profile.

## 2. Materials and Methods

### 2.1. Genome Sequencing

The plant material was provided by the Genebank of the National Institute of Agrobiological Sciences (NIAS). High-quality nuclear DNA with reduced organellar DNA was extracted from young leaves using a protocol designed for BAC DNA extraction with some modifications [10]. All sequencing reads were obtained using the Illumina HiSeq2000 at Operon Biotechnologies, Inc. (Eurofins Genomics). Standard short-read libraries and mate-paired libraries with 8 kbp insertion were built using the TruSeq SBS v5 for sequencing runs at 2 × 100 bp or 200 bp total. After sequencing, HiSeq Control Software v.1.4.8 and CASAVA 1.8.1 (Illumina) were utilized for base calling. Single-ended libraries and 3 kbp pair-ended libraries constructed with the GS FLX Titanium General Library Preparation Kit and Rapid Library Preparation Kit (Roche) were sequenced on Roche 454 FLX Titanium at the NIAS, and base calling was performed using the 454 FLX Titanium base caller.

### 2.2. Assembly and Reference Mapping

We constructed a* de novo* genome assembly (*G. max*_Enrei1) and reference genome assembly (*G. max*_Enrei2) to facilitate comprehensive analysis of the genome. The* G. max*_Enrei1 assembly was constructed from the Roche 454 FLX Titanium single-ended reads and pair-ended reads with 3 kbp insert, the Illumina HiSeq2000 pair-ended reads with 300 bp insert and mate-pair reads with 8 kbp insert, and the ABI 3730xl BAC-end reads using the Roche Newbler 2.7.

The* G. max*_Enrei2 assembly was derived from Roche 454 FLX Titanium single-ended reads and Illumina HiSeq2000 pair-ended reads were used for reference mapping with the BWA 0.7.5a (Li H. Aligning sequence reads, clone sequences, and assembly contigs with BWA-MEM, 2013; http://bio-bwa.sourceforge.net/), SAMtools 0.1.19 [11], and NIG script (NGS Surfer's wiki, http://cell-innovation.nig.ac.jp/wiki/tiki-index.php?page=samtools#mpileup_). The* G. max* cv. Williams 82, also referred to as Gmax275 genome assembly, was used for reference mapping. The pseudomolecules and scaffolds in the* G. max*_Enrei2 were searched for marker sequences by BLASTn (NCBI BLAST, ftp://ftp.ncbi.nih.gov/blast/). Then marker sequences were mapped in the regions with clear sequences, gap regions (indicated as N's in the sequence), BAC-end sequences (BES) hit position, marker hit position, and scaffold derived from* de novo* assembly hit position. Subsequently, the cutting points were identified to reconstruct the pseudomolecules and scaffolds.

### 2.3. Gene Models

The soybean parameter files were built from chromosome 16 region of hard masked Gmax275 genome [30,000,000–37,887,014 bps] using Augustus program [12]. The transposable elements in the scaffolds and pseudomolecules of the* G. max*_Enrei2 genome assembly were masked using RepeatMasker (Smit AFA, Hubley R., and Green P. RepeatMasker Open-3.0, 1996–2010, http://www.repeatmasker.org/) and the gene models were built using Augustus. These gene models were used as queries in BLASTn search using the soyTE as a database [13]. The filtered gene models with bit score of 100 and above were selected. Additionally, we used available RNAseq data (PRJDB3582) assembled by Trinity version 2014-07-17 [14]. A total of 172,753 gene models were extracted. For each gene, the longest ORF was identified using EMBOSS getorf [15].

### 2.4. Phylogenetic Analysis

The amino acid sequences of the gene models for* Arabidopsis thaliana* [16],* Arabidopsis lyrata* [17],* Medicago truncatula* [18], and* Oryza sativa* (annotation data on Os-Nipponbare-Reference-IRGSP-1.0, http://rapdb.dna.affrc.go.jp/download/archive/irgsp1/IRGSP-1.0_protein_2014-06-25.fasta.gz) were obtained. These were combined with the corresponding sequences of Gmax275 and* G. max*_Enrei2 for clustering with OrthoMCL v2.0.7 [19]. After removal of incomplete gene models, a set of single copy genes (Supplementary Table S1 in Supplementary Material available online at http://dx.doi.org/10.1155/2015/358127) was built from gene models that completely matched gene models in the genome and gene models derived from RNAseq. Then the fourfold degenerative sites derived from the refined single copy gene set were aligned using Clustal Omega 1.2.0 [20]. A guided tree was built by MEGA 6.06 [21] and the phylogenetic tree was constructed using PAML 4.8a [22], Multidivtime [23], and FigTree v1.4.2 (http://tree.bio.ed.ac.uk/software/figtree/).

### 2.5. Anthocyanin and Flavonoid Biosynthesis

All gene models in Gmax275 and* G. max*_Enrei2 associated with anthocyanin and flavonoid biosynthesis were extracted and clustered using OrthoMCL [19]. Then these gene models were associated by BLASTn.

### 2.6. Proteome Analysis

The proteome analysis of Enrei cultivar was performed using seeds. The cotyledons from ten seeds were grounded in liquid nitrogen and purified by phase separation using standard procedures [24]. The purified proteins were digested with trypsin. For mass spectrometry analysis, the eluted peptides were analyzed on a nanospray LTQ XL Orbitrap mass spectrometer and the MS spectra were used for protein identification. Identification of proteins was performed using the Mascot search engine version 2.4.1 (Matrix Science, London, UK) and Proteome Discoverer software version 1.4.0.288 (Thermo Fisher Scientific) against 54,175 soybean peptide sequences [7]. Mascot results were filtered with Mascot Percolator software to improve the accuracy and sensitivity of the peptide identification [25]. The protein abundance was analyzed using emPAI value as described in Shinoda et al. [26]. Furthermore, the protein gene models derived from Gmax275 and* G. max*_Enrei2 genome assemblies were associated using the clustered data obtained from OrthoMCL [19]. Additionally, these gene models were associated by BLASTn.

## 3. Results and Discussion

### 3.1. Genome Sequencing and Reference Mapping

The whole genome sequence of the Japanese soybean cultivar Enrei was assembled using a total of 22.2X coverage (Table 1). Reference mapping into the Gmax275 genome assembly was performed using DNA markers in the genetic linkage map of cultivar Enrei (Supplementary Table S2) and the* de novo* genome assembly (accession numbers BBNX01000001–BBNX01092182) (Supplementary Table S3). The ratio of* G. max*_Enrei2 to Gmax275 genome length (978,495,272 bps) [7] was 99.95%. The quality of the genome was evaluated by mapping marker sequences, BES, and the 56,264 gene models without alternative splicing in the Gmax275 genome [7]. As a result a total of 56,043 (99.6%) gene models in Gmax275 were represented in the Enrei genome. Additionally, a total of 1,860 marker sequences (Kaga et al., unpublished data) were mapped with a ratio of 98.8%. A total of 87 markers were unmatched in terms of linkage order and physical position (Supplementary Table S4). Additionally, a total of 92,451 BES pairs were mapped with a ratio of 76.3% (Supplementary Table S5). The Enrei genome sequence is deposited at the DNA Data Bank of Japan (DDBJ) under accession numbers BBNX02000001–BBNX02108601.

### 3.2. Gene Models

In total, 60,838 Enrei gene models were predicted (Table 1). Comparison with the gene models of Gmax275 [7] showed a longer mean coding sequence length (1,455 bps in Enrei and 1,168 bps in Gmax275) and a longer mean exon length (323 bps in Enrei and 231 bps in Gmax275). To complement the annotation of the genome sequence, gene models were mapped to the longest 172,753 open reading frame (ORF) sequences from the mRNAs of young leaves (Supplementary Table S6). In total, 11 gene models had no ORF hit sequences, 20,542 gene models had more than 50% coverage, 5,950 gene models had more than 90% coverage, and 2,269 gene models had 100% coverage.

As mean coding sequence length was 1,168.1 bps, mean number of exons per gene 5.0, and mean exon length 231.5 bps of 56,044 Gmax275 gene models without variant, Enrei number of exons per gene was shorter and CDS was longer. The difference in the gene models between Gmax275 and* G. max*_Enrei2 may be attributed to SNPs between the two cultivars as well as several parameters used in building the gene models.

### 3.3. SNPs and INDELs

A total of 1,659,041 SNPs and 344,418 insertions and deletions (INDELs) were identified between the* G. max*_Enrei2 and Gmax275 genome assemblies (Supplementary Table S7). Both SNPs and INDELs were largely predominant in chromosome 18 and relatively less predominant in chromosome 11. The average distance between SNPs was calculated at 589.8 bp/SNP against the Gmax275 genome assembly. The minimum average distance was 320.8 bp/SNP on chromosome 18 and maximum average distance was 984.9 bp/SNP on chromosome 5. Moreover, the total INDELs in Enrei represent approximately 50.79 Mbp of the genome sequence. These values however merely represent an overview of the differences in genome structure of the two cultivars. An accurate analysis of the SNPs and INDELs can be obtained only from a high-quality genome sequence of both cultivars.

### 3.4. Phylogenetic Analysis

**Figure 1:**
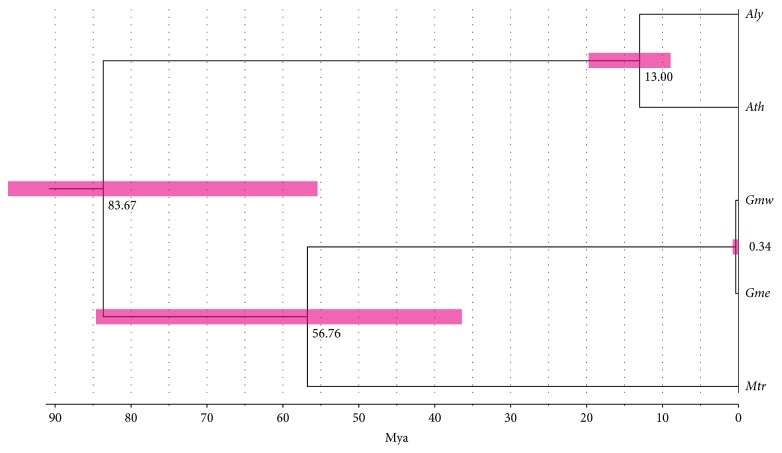
Phylogenetic tree of* G. max* cv. Williams 82 (*Gmw*),* G. max* cv. Enrei (*Gme*),* A. thaliana* (*Ath*),* A. lyrata* (*Aly*), and* M. truncatula* (*Mtr*). The pink bar represents the 95% probability density. Mya represents a unit in million years.

We applied OrthoMCL [19] to the clustered and aligned gene models of* A. thaliana* [16],* A. lyrata* [17],* G. max* cv. Williams 82 [7],* G. max* cv. Enrei,* M. truncatula* [18], and* O. sativa* (annotation data on Os-Nipponbare-Reference-IRGSP-1.0.). A set of filtered single copy genes was selected to calculate the phylogenetic relationships and divergence time among these species (Supplementary Table S1). Based on the phylogenetic divergence of* A. thaliana* which occurred about 13 Mya [27], the divergence between* G. max* cv. Williams 82 and cv. Enrei was estimated at 0.34 Mya (95PD: 0.78–0.10 Mya), much later than the calculated divergence time between* A. thaliana* and* A. lyrata* (95PD: 19.30–8.52 Mya; Figure 1). The divergence between the* Glycine* clade and* M. truncatula* was estimated to have occurred around 56.76 Mya (95PD: 84.54–36.99 Mya). On the other hand, the divergence between the* G. max*/*M. truncatula* clade and the* Arabidopsis* clade must have occurred much earlier around 83.67 Mya (95PD: 122.51–55.57 Mya). Previous studies have shown a divergence time of 54 Mya between* M. truncatula* and the* Glycine* clade [28]. A whole genome duplication (WGD) which occurred around 58 Mya had been a major factor in shaping the* M. truncatula* genome [18].

The complex of* G. max* and its wild relative,* G. soja,* diverged from the most recent common ancestor around 0.27 Mya [4] or 0.8 Mya [5]. Assuming that* G. max* diverged from* G. soja* at around 0.8 Mya, the divergence of the branch for both Williams 82 and Enrei at around 0.34 Mya was much later than previously estimated. As Li et al. [5] pointed out, divergent selection may have contributed to the differentiation of* G. soja* and* G. max* before domestication of* G. max*. The divergent selection as adaptation to different environments must have contributed to the differentiation of both cv. Williams 82 and cv. Enrei from the most recent common ancestor.

### 3.5. Anthocyanin and Flavonoid Biosynthesis

**Figure 2:**
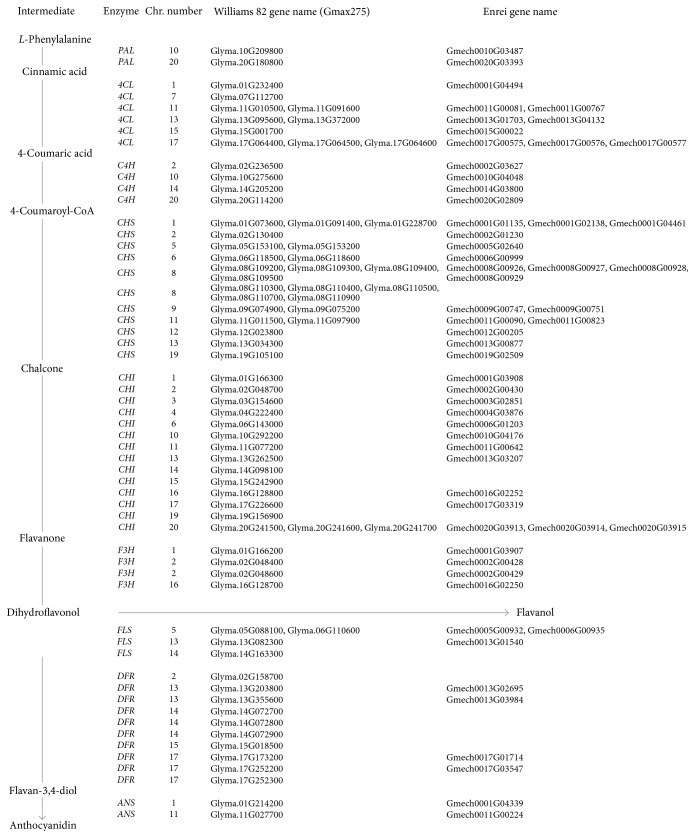
Enzymes involved in the major pathway for anthocyanin and flavonoid biosynthesis and the corresponding genes in Gmax275 and* G. max* cv. Enrei.* PAL* (phenylalanine ammonia-lyase),* 4CL* (4-coumaroyl-CoA-ligase),* C4H* (cinnamate-4-hydroxylase),* CHS* (chalcone synthase),* CHI* (chalcone isomerase),* F3H* (flavanone 3-hydroxylase),* FLS* (flavonol synthase),* DFR* (dihydroflavonol 4-reductase), and* ANS* (anthocyanidin synthase).

**Figure 3:**
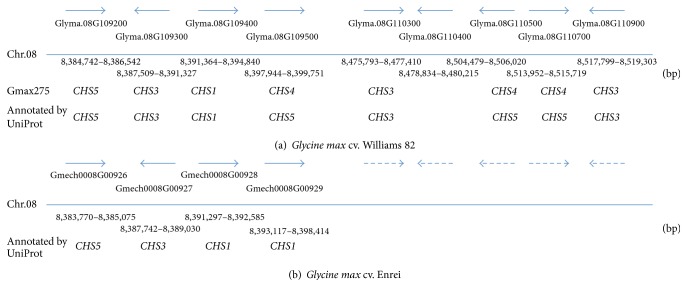
A region in soybean chromosome 8 showing the position of* CHS* gene clusters. The region encompassing 8.3~8.5 Mb of soybean chromosome 8 of Williams 82 Gmax275 (a) and Enrei cultivar (b) is characterized by* CHS* gene clusters. Most of the* CHS* genes correspond in both cultivars as indicated by the position and UniProt annotation of identified genes. However many* CHS* genes could not be localized in Enrei cultivar due to fragmented sequence.

In soybeans, several chalcone synthesis genes, namely,* CHS3* (P19168),* CHS1* (P24826),* CHS7* (P30081),* CHS4* (Q6X0N0), and* CHS8* (AY237728), are associated with seed coat pigmentation [29]. The physical position of these* CHS* genes was determined using BAC assembly [30, 31] for loci associated with RNA silencing and WGS assembly [7]. The corresponding genes in the Gmax275 genome assembly are as follows:* CHS1* (Glyma.08G109400),* CHS2* (Glyma.05G153200),* CHS3* (Glyma.08G110300 and Glyma.08G110900),* CHS4* (Glyma.08G110500 and Glyma.08G110700),* CHS5* (Glyma.08G109200, Glyma.08G109300, and Glyma.08G110400),* CHS6* (Glyma.09G075200),* CHS7* (Glyma.01G228700),* CHS8* (Glyma.11G011500), and* CHS9* (Glyma.08G109500). Most of the genes in the pathway were commonly represented in both cultivars (Figure 2). However, one 4CL gene in chromosome 7, 5* CHS* genes in chromosome 8, 3 CHI genes in 14, 15, and 19, respectively, 1 FLS gene in chromosome 14, and 6 DFR genes on chromosomes 2, 14, 15, and 17 were not found in the Enrei genome. Most of the* CHS* genes correspond in both cultivars as indicated by the position and UniProt annotation of identified genes except for those genes that could not be localized in the Enrei cultivar due to fragmented sequence (Figure 3). As most of these genes are involved in pigmentation of seed and hilum color in soybean, the absence of these genes in the Enrei genome in relation to anthocyanin and flavonoid biosynthesis pathway may be associated with gene silencing due to siRNA activity [32, 33].

### 3.6. Protein

Using gene models associated with seed proteome data, a total of 164 protein gene models corresponding to storage proteins, lipid synthesis/degradation enzymes, sorting/folding-related proteins, late embryogenesis abundant (LEA) protein, glycolysis pathway enzymes, protease/protease inhibitors, and others were identified (Supplementary Table S8). The protein content of dry seeds was 35–42% [34, 35] of the dry weight, and 70% of protein consists of 7S and 11S globulins [34, 36], which are part of the cupin superfamily (http://www.ebi.ac.uk/interpro/entry/IPR006045), corresponding to beta-conglycinin and glycinin, respectively [37]. To identify the proteins associated with grain filling of soybean seeds, we conducted a proteome analysis of the cotyledon. A total of 160 protein gene models in Gmax189 correspond to Enrei protein gene models ranging from 7.87 mol% to 0.03 mol% (Supplementary Table S8). Most of these proteins are storage proteins and cupin including beta-conglycinin and glycinin representing about 42% of total mol% and about 55% of total weight (sum of mass *∗* mol) (Table 2). Genes controlling the content of seed storage proteins were also highly represented [38, 39]. Genes associated with lipid metabolism such as lipoxygenase 1, peroxygenase 2, and oleosin family protein genes [40]; gene associated with sorting/folding-related protein such as HSP20-like chaperone, PDI-like, SNF7 family, and vacuolar sorting receptor proteins; and LEA protein genes which may be important in protecting other proteins from aggregations were highly represented in the Enrei genome. In addition, some genes involved in glycolysis pathway, enzymes, and proteinase/protease inhibitors, which may play an important role in germination stage, were also found. This proteome profile may provide the basis for understanding cultivar diversity and adaptation to cultivation condition.

### 3.7. Enrei Genome Database

All sequencing data can be accessed in DAIZUbase (http://daizu.dna.affrc.go.jp/enrei/), an informatics resource for soybean genomics focusing on the Japanese soybean cultivar Enrei. The database is provided with a GBrowse [41] with interactive pages for displaying the Enrei genome sequence as well all aligned Enrei BAC clones and accompanying annotations. DAIZUbase also includes a unified map, which indicates the relationship between the linkage map and the physical map of the Enrei cultivar.

## 4. Conclusion

The genome sequence of the Japanese cultivar Enrei will provide valuable information for improvement of soybean cultivars adapted to domestic cultivation. The genome sequence will complement emerging strategies for effective soybean breeding through analysis of the genome structure of Japanese (domestic) soybean, development of DNA markers serving as landmarks of agronomically important traits, development of research resources for the identification of important genes in soybean, and isolation of genes controlling important traits such as disease and pest resistance, productivity, and regional adaptability. Detailed knowledge of the genes controlling specific traits will allow for more efficient soybean improvement enabling researchers to develop plant types adaptable to various environmental conditions.

## Supplementary Material

The supplementary materials provide details of reference mapping, genomic and phylogenetic analysis of *Glycine max* cv. Enrei such as the filtered set of single copy genes used for phylogenetic analysis based on OrthoMCL clustering (Table S1), a list of DNA markers derived from cv. Enrei used for reference mapping (Table S2), details of the genome sequence reads used for reference mapping and de novo assembly (Table S3), a list of markers with unmatched linkage order and physical position (Table S4), the BAC-end sequences (BES) mapped in the cv. Enrei genome assembly (Table S5), the sequencing and assembly of cDNA libraries derived from young leaves (Table S6), the SNPs and INDELs in *G. max* cv. Enrei based on Gmax275 genome assembly (Table S7), and the seed proteome data and corresponding models in Gmax275 and cv. Enrei (Table S8).

## Figures and Tables

**Table 1:** Genome assembly and annotation of *G. max* cv. Enrei.

Reference mapping length	With gaps [bp]	Without gaps [bp]	Ratio
Chromosome	946,877,581	904,901,085	95.6
Scaffold	31,116,190	22,803,649	73.3
Total	977,993,771	927,704,734	94.9

Gene models			

Number of gene models	60,838		
Mean coding sequence length	1455.3 [bp]		
Mean number of exons per gene	4.5		
Mean exon length	323.4 [bp]		

**Table 2:** Composition of storage proteins in *G. max* cv. Enrei.

Chromosome	Related number of gene	Weight % (mass *∗* mol)	mol %
Chr10	6	19.8	14.27
Chr20	4	15.7	14.58
Chr03	1	6.6	4.31
Chr13	1	4.6	3.06
Chr19	1	2.8	1.88
Chr04	1	2.4	1.99
Chr02	1	2.1	1.36
Chr11	1	1.2	0.85
Chr01	1	0.1	0.07

Total	17	55.4	42.4
